# Comparison between Intramuscular Multichannel Electrodes and Supramysial Multichannel Electrodes via EMG Measurements for Potential Use as Larynx Stimulation Electrodes: In Vivo Animal Analysis

**DOI:** 10.3390/s19204477

**Published:** 2019-10-16

**Authors:** Bernd Faenger, Nikolaus P. Schumann, Christoph Anders, Dirk Arnold, Roland Grassme, Orlando Guntinas-Lichius, Hans-Christoph Scholle

**Affiliations:** 1 Division for Motor Research, Pathophysiology and Biomechanics, Department for Trauma-, Hand- and Reconstructive Surgery, Jena University Hospital, Friedrich-Schiller-University Jena, 07743 Jena, Germany; Nikolaus-P.Schumann@med.uni-jena.de (N.P.S.); Christoph.Anders@med.uni-jena.de (C.A.); D.Arnold@uni-jena.de (D.A.); Roland.Grassme@med.uni-jena.de (R.G.); Hans-Christoph.Scholle@med.uni-jena.de (H.-C.S.); 2 Institute of Diagnostic and Interventional Radiology, Department of Experimental Radiology, Research Center Lobeda, Jena University Hospital, Friedrich-Schiller-University, 07747 Jena, Germany; 3 Institute of Pathology, Neuropathology Section, Jena University Hospital, Friedrich-Schiller-University, 07747 Jena, Germany; 4 Institute of Zoology and Evolutionary Research, Friedrich-Schiller-University Jena, 07743 Jena, Germany; 5 Employer’s Liability Insurance Association for Food and the Hospitality Industry (Berufsgenossenschaft Nahrungsmittel und Gastgewerbe), Department for Prevention, branch office Erfurt, 99097 Erfurt, Germany; 6 Department of Otorhinolaryngology, Jena University Hospital, 07747 Jena, Germany; Orlando.Guntinas@med.uni-jena.de

**Keywords:** electromyography, functional electrical stimulation, larynx pacemaker, multichannel array electrodes, supramysial multichannel electrodes

## Abstract

One of the most common causes for larynx paralysis is the injury of the recurrent laryngeal nerve which, among others, causes the paralysis of the posterior cricoarytenoideus muscle (PCA). Electrical stimulation of PCA offers an approach to retaining the function of the paralyzed larynx muscle. The study aim was to test the applicability of an intramuscular multichannel array electrode as a measuring electrode for myoelectrical potentials and as a possible electrode for stimulation, e.g., posterior cricoarytenoideus muscle stimulation. For this purpose, two different kinds of electrodes were compared. 42 intramuscular multichannel array electrodes and 11 supramysial multichannel electrodes were implanted into the triceps brachii muscle of rats. The triceps brachii muscle of rats is suitable to serve as a substitute muscle for the human PCA muscle in an in vivo animal model. It has the same striated muscle cells, is of comparable size, and fundamentally serves a similar function to the human PCA muscle during normal respiration. Walking and breathing are circular functions that cause minimal muscle fatigue when carried out steadily. In total, the myoelectrical activity of 6703 steps could be recorded, allowing a comparison and statistical analysis of the EMG amplitudes and EMG activation patterns. Small differences can be detected between the EMG signals of both electrode types which, however, can be explained physiologically. Both electrode types reveal the basic characteristics of the triceps brachii muscle activity, namely the muscle contraction strength and the coordination pattern. This indicates that the intramuscular electrode may be applied for a detailed analysis of the human larynx.

## 1. Introduction

The paired posterior cricoarytenoid (PCA muscle) in humans is the only muscle that is responsible for the opening of the glottis [1,2]. The paralysis of this muscle causes a disturbed voice function, aspiration and shortness of breath. A “laryngeal pacemaker” would revitalize the glottis motor function, i.e., lead to the opening and closing of the vocal cords and an associated adjustment of the glottis [2,3]. Electrical stimulation could serve as a temporary measure to maintain muscle strength (contractility) and motility of the paralyzed laryngeal muscles to onset of spontaneous reinnervation, but also as a permanent solution in case of non-occurrence of reinnervation.

In practice, the laryngeal EMG of the PCA muscle serves to determine the innervation condition of the larynx in case of vocal cords paralysis. EMG measurements allow to draw conclusions about the causes of the vocal cords paralysis. The myoelectrical activity of the inner larynx muscles is measured with the patient awake and under local anesthesia, generally induced endolaryngeally via bipolar hooked-wire electrodes [4,5,6] and in some cases transcutaneously via the ligamentum conicum, using a concentric bipolar needle electrode.

Since an electrode must be implanted to achieve electrostimulation, it would be appropriate to use an electrode which can also measure myoelectrical activity. This way, it is possible to conduct a detailed study of the course of the experiment, enabling, for instance, the detection of a potential reinnervation over time. Using the multichannel array electrode, several channels may be recorded simultaneously. By these means, the electrode allows a sort of mapping. The EMG characteristics may be consulted for detailed evaluations of the PCA innervation condition, as well as for later improvement of the stimulation parameters based on the new EMG data available.

For an electrode for chronic stimulation of the PCA muscle, the biocompatibility of the materials used is very important. Due to their material (silver) and design (surface electrode), the established in animal supramysial multichannel electrodes cannot be used as an EMG measurement and stimulation electrode for the posterior cricoarytenoid muscle of the human larynx. With this type of electrode there is silver corrosion of the electrode surfaces. In addition, the difficult anatomical position of the PCA muscle prevents the attachment of a supramysial multichannel electrode. An atraumatic intramuscular array electrode therefore represents a possible alternative. This intramuscular multichannel array electrode was described recently [7]; its suitability for use as stimulation electrode is proven and the best points for muscle stimulation have been defined [8]. Eight array electrodes were implanted in the triceps brachii muscle of four rats. The animal model identified the stimulation points for minimal possible muscle fatigue stimulation as being located close to the points of entrance of the nerve into the muscle.

In order to test the intramuscular electrode for suitability in EMG measurement, the EMG signals in this work also were measured from the triceps brachii muscle of rats. There are several reasons why the triceps brachii muscle of rats is suitable to serve as a substitute muscle for the human PCA muscle in an in vivo animal model. It has the same striated muscle cells, is of comparable size, and fundamentally serves a similar function to the human PCA muscle during normal respiration and, partially, even functions observed during phonation. Walking and breathing are circular functions that cause minimal muscle fatigue when carried out steadily. Both the PCA muscle and the triceps brachii combine a fine-motor activation with a temporary high strength development. To provide further explanations for the comparability of the measurement results at the triceps brachii and the expected results for the PCA muscle, the physiological functions of the two triceps brachii muscle bellies during rat locomotion are defined in the discussion. The use of the triceps brachii in these experiments is also beneficial due to its accessibility as compared to other possible in vivo animal models, e.g., the analysis of a guinea pig’s larynx [9].

Both intramuscular array electrodes and the established supramysial electrodes were implanted to allow a later comparison of the results. Due to the similar design (multichannel electrodes) we expected that both electrode types generally show the changes in the amplitude level and amplitude pattern of the triceps brachii muscle during movement.

## 2. Materials and Methods

### 2.1. Subjects

The experiments were performed on 32 female adult rats (Wister strain). All animals were kept according to the German animal welfare regulations. The experiments were registered and approved by the Committee for Animal Research of the Freistaat Thuringen, Germany (Reg.-Nr.: 02-27/05).

### 2.2. Surgery

**Figure 1:**
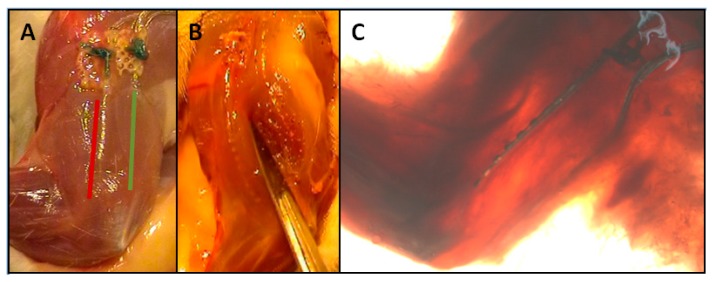
Left foreleg of rat with intramuscular array electrodes positions. (**A**,**B**) Incision, GREEN: Intramuscular array electrode in the c. longum, RED: Intramuscular array electrode in the c. laterale, (**C**) Nerves stained using Sihler’s staining technique.

A detailed description of the implantation of intramuscular array electrodes was published recently [7]. Briefly speaking, two intramuscular array electrodes were applied into the M. triceps, one in the caput (c.) longum and the other one in the caput (c.) laterale (Figure 1). The supramysial surface electrodes were fastened to the muscle belly using single button sutures. For these electrodes, all electrode surfaces lay in a common silicone substrate which was positioned enabling half of the electrode surfaces to record the activities of the c. longum and the other half those of the c. laterale.

### 2.3. Electrodes

**Figure 2:**
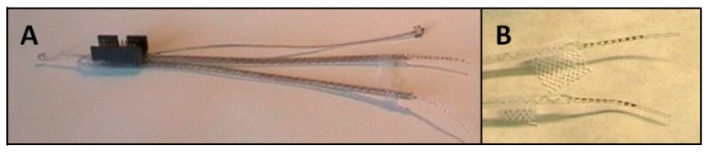
Intramuscular array electrodes. (**A**) Two intramuscular array electrodes with connector, (**B**) Electrode tips with electrode plates.

To ensure the measurement of the myoelectrical activity of the two triceps brachii muscle parts caput longum and caput laterale, an intramuscular array electrode (MED-EL^®^, Innsbruck, Austria) was used (Figure 2). A description of the characteristics of this electrode is described elsewhere [7]. The wires and tips were composed of platinum iridium, Teflon insulation-coated single wires, and electrode surfaces were embedded in silicone-except for an area measuring 1 mm (= electrode diameter). The distance of the electrodes (the distance of the electrode surfaces’ centers to each another) amounts to 1.5 mm. Nine electrode surfaces were placed onto the electrode tip which was casted in silicone and had a total length of approximately 15 mm. The lead wires of two array electrodes were, respectively, connected to a micro-connector (ODU^®^, Mini-Fix^®^, Mühlendorf am Inn, Germany). Additionally, two reference electrodes were connected. The two reference electrodes were positioned away from muscle (base of the tail and skull). A total of 42 electrodes were implanted. 42 array electrodes were used for 21 animals. One electrode per animal to measure the c. longum and one for measuring the c. lateral.

**Figure 3:**
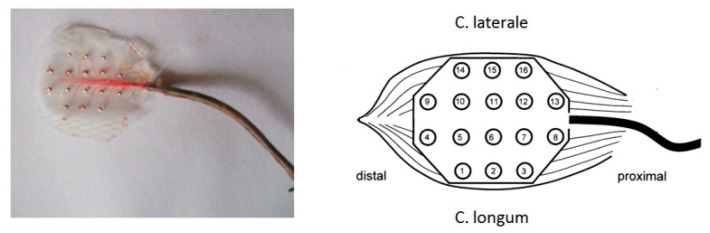
Silber grid electrode for supramysial use.

Further electrodes used were custom-made supramysial surface grid-electrodes, build at the Friedrich-Schiller-University’s central workshop in Jena [10] (Figure 3). Altogether, 11 electrodes of this supramysial surface grid-electrode type were implanted. Each electrode had 16 silver ball electrode surfaces, the balls being cast in silicone leaving unsealed the front side which got placed onto the muscle. The diameter of the semi-spherical electrode surfaces used to measure the EMG patterns amounted to approximately 0.4 mm. The distance of the electrode surfaces’ centers to each another amounted to 3 mm. The lead wires were made of silver wire with a 0.75 µm diameter. The additional reference electrode was formed from the silver wire strand of its lead. At the end of the supply line, a round loop with a diameter of approx. 3 mm was formed. The reference electrode was fixed onto the animal’s backs. All lead wires were isolated separately and then jointly covered by a flexible steel spring to prevent bending. The micro-connector used with all other electrode types (ODU^®^, Mini-Fix^®^, 20 pins) was soldered to the end of all lead wires.

Only 11 electrodes were produced. Because of their design and material, they cannot be used for laryngeal application. A direct further development of this electrode would not have been successful. 11 supramysial surface grid electrodes correspond to 22 intramuscular array electrodes due to their design. These electrodes were positioned so that half of the electrode surfaces could record the activities of the c. longum and the other half the activities of the c. laterale.

### 2.4. EMG Measurement and High-Speed Videography

**Figure 4:**
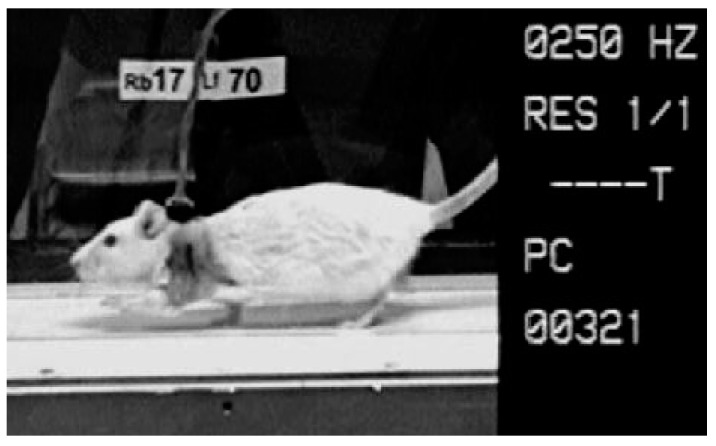
Rat connected to the EMG amplifier via nape plug. The animal is walking on a motorized treadmill.

A 16-channel EMG system (16-channel EMG system Biovision, Wehrheim, Germany; 10–700 Hz, sampling rate 4000 Hz) was used to measure the electromyographic activation patterns of the muscular system. The EMG was recorded monopolarly, using software provided by GJB (GJB Datentechnik GmbH^®^, Langewiesen, Germany), and while the animals were walking over a treadmill (Figure 4).

The use of the Camsys^®^ high-speed camera system provided by Mikromak^®^ (Erlangen, Germany) enabled the synchronous videography of the animals during EMG measurement, registering their movements with a rate of 250 images per second.

### 2.5. EMG Analysis

**Figure 5:**
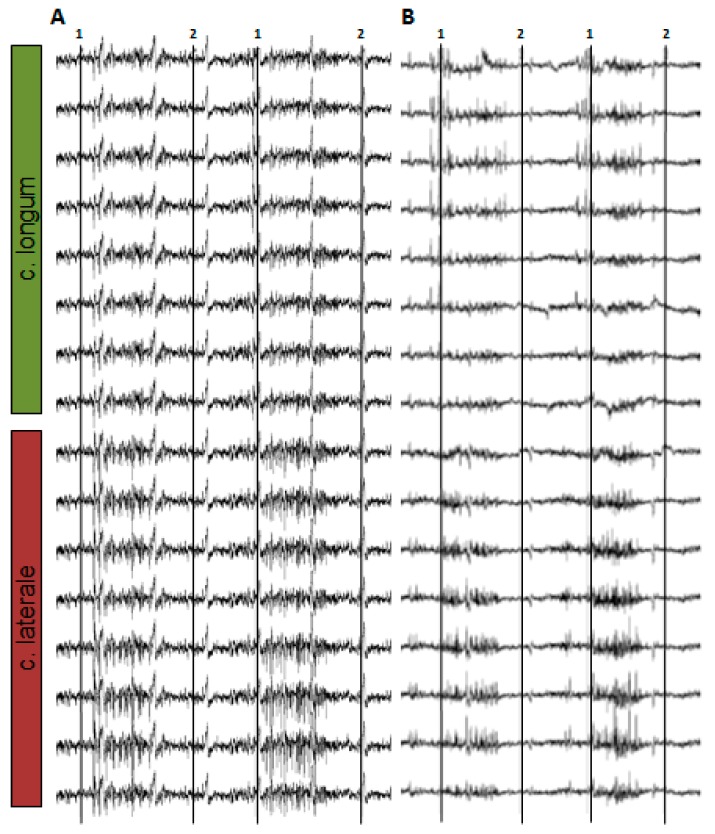
Exemplary representation of monopolar EMG signals during locomotion on treadmill. (**A**) 16 channel; grid electrodes. (**B**) 16 channel; array with the row of 8 electrodes. 1: foot-down marker. 2: foot-up marker.

The foot-down and foot-up points were determined visually via the high-speed videos which were synchronized with the EMG. Figure 5 shows an example of the monopolar EMG signal while a rat is running on a treadmill.

**Figure 6:**
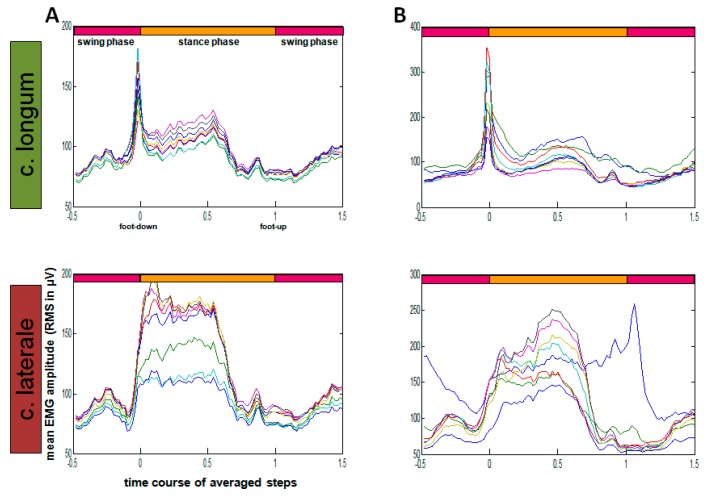
Exemplary representation of monopolar EMG, mean EMG amplitude (RMS) of time-normalized steps. All step duration’s were normalized to 100 points. Each color represents a channel of average steps of one rat. (**A**) 16 channel; grid electrodes; one animal with 587 steps. (**B**) 16 channel; array with the row of 8 electrodes; one animal with 935 steps.

RMS calculation (root mean square, sliding window 10 ms) was executed via Matlab^®^ (The MathWorks^®^, Inc., Natick, MA, USA) (Figure 6).

In order to allow comparison of steps with different lengths, time normalization was introduced. Reference duration was the stance phase between the up (completed lift of the foot from the floor) and down movements (hard floor contact). Applying an EMG interval half the duration of the stance phase, the preceding and following swing phases were considered. The time-normalized amplitude pattern refers to 100 relative time points, whereas the ratio of the preceding swing phase parts is determined to be 50:100:50, considering the total stance phase and the succeeding swing phase.

A standardization of the waves was necessary in order to allow a direct comparison of the two muscle bellies’ activation behavior (c. longum und c. laterale) [11]. Standardization was used to balance the inevitable interindividual differences between amplitudes. Standardization of the amplitude values was performed according to the respective maximum of each step cycle. In the following, these normalized EMG-amplitude courses will be specified as “amplitude patterns” or “coordination patterns” to distinguish them from not standardized amplitude courses.

### 2.6. Statistics

The following testing methods were applied:

(1) The EMG data of the respective muscle belly was tested for reliability [12].
(2) The EMG data was checked for normal distribution between the animals (Kolmogorov-Smirnov test). Since the data was not distributed normally according to the Kolmogorov-Smirnov test, only tests for not normally distributed data (non-parametrical tests) are used in the following points.
(3) The data of c. longum and c. laterale was tested for significant differences using the Wilcoxon test for paired samples. If the EMG data is significantly different, it must be concluded that both muscle bellies have different activation patterns.
(4) The data obtained for c. longum was tested for significant differences (significance level was *p* < 0.05) in intramuscular array electrodes and supramysial surface electrodes using the Mann–Whitney U test. The latter can be used to determine whether the amplitude and amplitude patterns of the two electrode types’ EMGs may be compared.
(5) The data obtained for c. lateral was tested for significant differences (significance level was *p* < 0.05) for intramuscular array electrodes and supramysial surface electrodes using the Mann–Whitney U test in order to determine whether the EMGs of the two electrode types are comparable in amplitude and amplitude pattern.

All diagrams in this article show statistical tests which are represented in combination with curves depicting the EMG amplitude course as a representative curve of all eight electrode surfaces of the c. longum and c. laterale muscle bellies during the time-normalized average steps. The mean EMG amplitude representation serves to compare the amplitude levels. Differences within the amplitude patterns or rather coordination patterns become clear through depiction of the relative EMG amplitude.

## 3. Results

### 3.1. EMG Analysis

The first statistical test conducted was to determine to what extent the eight electrode surfaces of each muscle belly coincide with respect to the normalized step. The tested parameters are highly reliable, having a correlation coefficient of *r* > 0.9 [12]. This result permitted to mean the values of eight electrode surfaces for each muscle belly.

### 3.2. Comparison of Relative and Mean EMG Amplitude between Muscle Bellies

**Figure 7:**
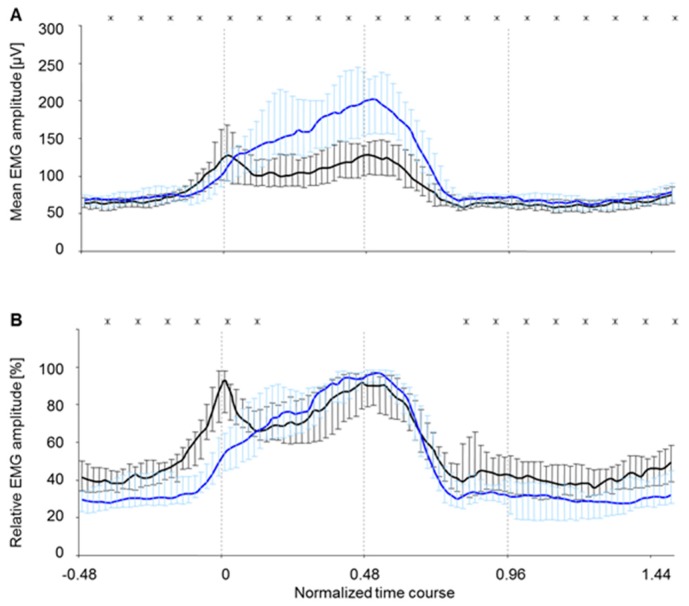
EMG amplitude as representative curves of all eight electrode surfaces of the two muscle bellies c. longum and c. laterale during the time-normalized steps. Values of both electrode types are jointly displayed: intramuscular array electrode (5903 steps) and supramysial surface electrode (800 steps). They are represented as median ± quartile. The significant difference is represented according to Wilcoxon (x: *p* < 0.05) for 5% of each normalized time course. (**A**) Mean EMG amplitude (RMS), (**B**) Relative EMG amplitude, black line = c. longum, blue line = c. laterale, 0–0.96 Stance phase, 0 foot-down point, 0.48 Midpoint of stance phase, 0.96 foot-up point.

During locomotion on the treadmill, the two muscle bellies c. longum and c. laterale of the triceps brachii muscle of rats showed significant differences concerning their EMG amplitude level (time-normalized step cycle, Figure 7A). The EMG amplitude pattern (time-normalized step cycle, Figure 7B) of the muscle bellies c. longum and c. laterale revealed representative differences during the swing phase. During stance phase, the amplitude patterns only differed significantly in the first and last quarter. In total, the level of the c. longum lay clearly below that of the c. laterale. Dispersion, represented by quartiles, was much lower for c. longum (Figure 7A) than for c. laterale. In the amplitude-normalized curves (Figure 7B), dispersion was almost equal for both muscle bellies. This means that amplitude differences were conditioned by individually varying activation levels, whereas the amplitude patterns were, in contrast, robust. Amplitude patterns were interindividually more stable than the amplitude levels.

### 3.3. Comparison of EMG Amplitude Levels and Muscles Coordination Pattern between the Two Electrode Types for the c. longum

**Figure 8:**
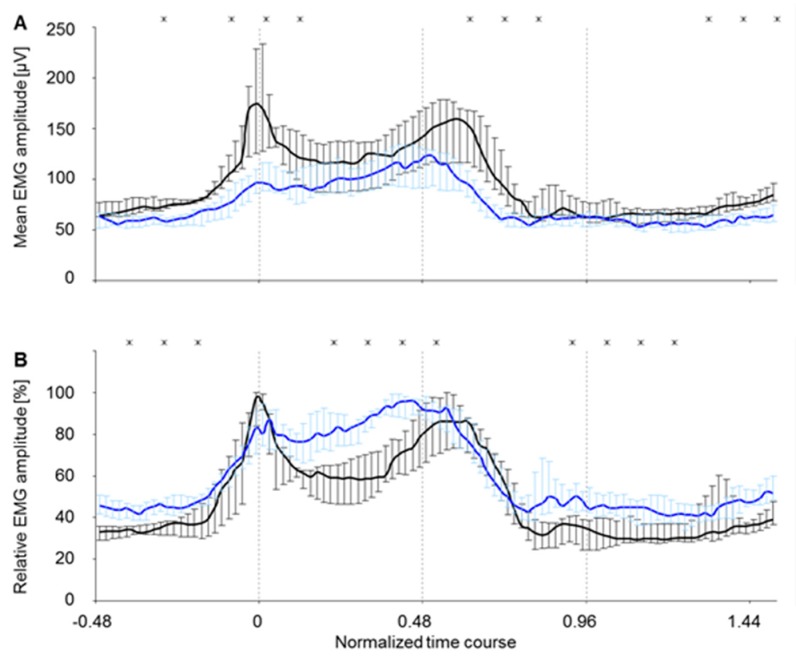
EMG amplitude as representative curve of all eight electrode surfaces of c. longum during the time-normalized average steps. Values of electrode types are displayed separately: intramuscular array electrode (5903 steps) and supramysial surface electrode (800 steps). They are represented as median ± quartile. The significant difference is represented according to U test (x: *p* < 0.05) for 5% of each normalized time course. (**A**) Mean EMG amplitude (RMS), (**B**) Relative EMG amplitude, black line = c. longum measured via array electrodes, blue line = c. longum measured via surface electrodes, 0–0.96 Stance phase, 0 foot-down point, 0.48 Midpoint of stance phase, 0.96 foot-up point.

When directly comparing the RMS curves (c. longum) for all animals measured by means of an intramuscular array electrode and those having been implanted a supramysial surface electrode, it shows that the mean amplitude of all supramysial surface electrodes was lower than the amplitude of all intramuscular array electrodes for nearly the whole step duration (Figure 8A). Significant differences can be noted for the foot-down movement and for the falling flank after the second maximum. A comparison of the relative EMG amplitudes, namely the amplitude patterns for both electrode types, revealed similar basic characteristics for both electrode types (Figure 8B). The muscle belly was activated even before the point in time “0” (hard floor contact). Both curves showed a maximum close to the point in time “0”, where the supramysial surface electrodes presented a small double peak. Following this maximum, the measured EMG amplitude of the animals measured via supramysial surface electrodes fell to a lesser extent than the one measured by intramuscular array electrodes. After passing through a minimum, both electrode types register a slow increase of the amplitude during the stance phase until reaching a new maximum. In case of the array electrodes, the first maximum reached was higher than the second one. For the supramysial surface electrodes, in contrast, the second maximum was higher than the first one. In addition, the supramysial surface electrodes reached their second maximum even before getting to the midpoint of the stance phase (point in time “0.48”), whereas the intramuscular array electrodes registered their second high shortly after. Having recorded the second maximum, signal amplitude dropped slowly for both electrode types until reaching the minimal level just before the end of the stance phase. With exception of pre-activation, this level was held for the duration of the swing phase. Both curves in Figure 8B depicted a similar steepness in increase and decrease for the c. longum at the beginning and the end of activation. There were no significant differences between the amplitude patterns when considering the moment the foot touches the ground or the descending flank following the second maximum (U test). However, significant differences were registered for the rising flank which precedes the second maximum and during swing phase.

### 3.4. Comparison of EMG Amplitude Levels and Muscles Coordination Patterns between the Two Electrode Types for the c. laterale

**Figure 9:**
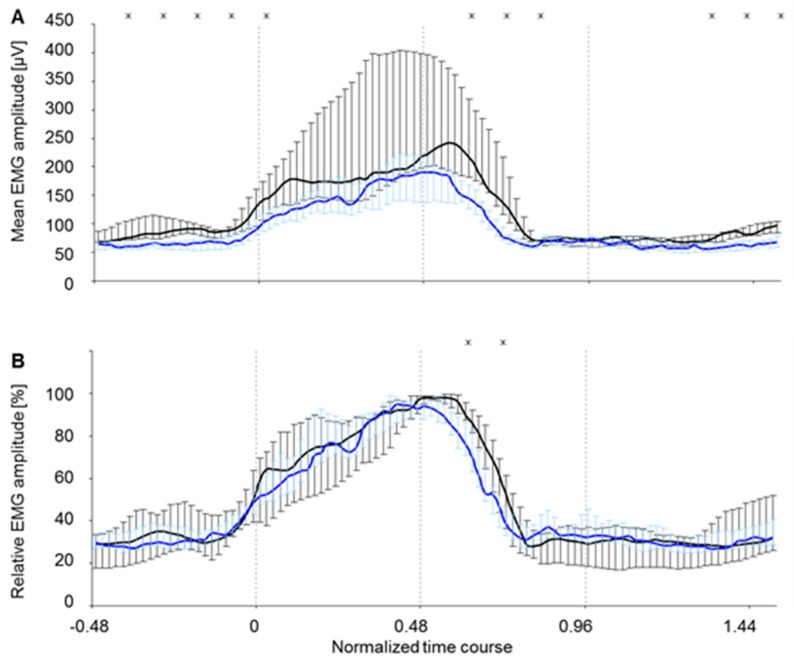
EMG amplitude representative curve of all eight electrode surfaces of c. laterale during the time-normalized average steps. Values of electrode types are displayed separately: intramuscular array electrode (5903 steps) and supramysial surface electrode (800 steps). They are represented as median ± quartile. The significant difference is represented according to U test (x: *p* < 0.05) for 5% of each normalized time course. (**A**) Mean EMG amplitude (RMS), (**B**) Relative EMG amplitude, black line = c. laterale measured via array electrodes, blue line = c. laterale measured via surface electrodes, 0–0.96 Stance phase, 0 foot-down point, 0.48 Midpoint of stance phase, 0.96 foot-up point.

A direct comparison of the RMS curves (c. laterale) of all animals measured by means of an intramuscular array electrode and of those having been implanted a supramysial surface electrode showed that the mean amplitude of the supramysial surface electrodes was lower than the intramuscular array electrodes’ amplitude for nearly the whole step duration (Figure 9A). There were significant differences in the falling flank which follows the second maximum. When comparing the relative EMG amplitudes, namely the amplitude patterns for both electrode types, similar basic characteristics for both electrode types were revealed (Figure 9B). The muscle belly was activated even before the point in time the foot touches the ground completely. The amplitude was rising steadily until reaching the approximate midpoint of the stance phase (point in time “0.48”). In case of the supramysial surface electrodes, the absolute maximum was reached just before the point in time “0.48”, whereas it is exactly “0.48” for the intramuscular array electrodes. After this maximum, the amplitude of both electrode types rapidly fell to its basic level, the supramysial surface electrodes’ potential dropping an instant earlier than that of the intramuscular array electrodes. This rising flank revealed significant differences between the electrodes. The basic level was reached almost simultaneously by both electrode types. Except for pre-activation, this level was held approximately for the duration of the swing phase. When comparing the EMG data of both electrode types for the c. laterale, statistical evaluation showed that the RMS curve amplitudes for intramuscular array electrodes did not list significant differences starting from the moment the foot touches the ground until the mid-stance phase. Severe dispersion of the amplitude values, which are represented by quartiles, was the result of the individually varying action potentials of each animal. The analysis of the relative EMG amplitudes (calculated from the median EMG values) showed that the amplitude patterns for both electrode types are nearly identical.

## 4. Discussion

The main result is that both electrode types basically show the changes in amplitude and activation pattern of the triceps brachii muscle during movement. The measured values of both electrode types clearly show the typical activation patterns of both muscle bellies when rats walk on a treadmill.

When checking the EMG amplitudes and EMG activation patterns of the two triceps brachii muscle bellies c. longum and c. laterale for significant differences, the results of Scholle et al., who detected significant differences in the EMG amplitudes of the two muscle bellies when using a supramysial surface electrode [13], could be confirmed. The EMG amplitudes of the muscle bellies, measured by both supramysial surface electrodes and intramuscular array electrodes, also displayed significant differences for the whole step duration.

During the second and third quarter of the stance phase, however, the amplitude patterns of the muscle bellies were not significantly different from each other. This indicates that both muscles have the same physiological function during this phase.

It can be expected that the different areas of the PCA muscle also have to serve different functions temporarily, depending on whether it focusses on the opening and closing of the rima glottis during respiration without phonation or whether at the same time, at least temporarily or partially, it is used for joint stability.

Until now, the c. longum has been declared to be an antigravity muscle [14]. The present study considers the c. longum to be a muscle ensuring primarily joint stability and joint adjustment, and the c. laterale to be an antigravity muscle. The c. longum evidently serves as a joint stabilizer when the foot touches the ground. As revealed by the not significantly different amplitude patterns of the two muscle bellies, it then supports the c. laterale in its function as antigravity muscle during the rest of the stance phase. It can be assumed that some areas of the PCA muscle are used for joint adjustment and joint stabilization, while other areas primarily counteract the forces of the opponent muscles when opening the Rima glottidis. Depending on the region and time range in which the PCA muscle is measured, an EMG activity pattern corresponding to the c. longum or c. lateral activity pattern would be expected. All through the swing phase, the c. longum is significantly more active than the c. laterale, which is a further indication of its function as a muscle guaranteeing joint stability and joint adjustment. Joints are also secured in their position during swing phase.

The significant differences noted in the EMG amplitudes for both electrode types in the c. longum result from the fact that an intramuscular array electrode is positioned closer to the active muscle fiber than a supramysial surface electrode. It has been noted by Grassme et al. [15] that the activation patterns recorded by supramysial surface electrodes originate in the interior of the muscle belly. The c. longum is further likely to be the muscle belly whose prime responsibility is joint stability. Therefore, this muscle must be steered at in a fine and differentiated manner [13,15,16,17]. Such fine control can also be expected for the control of the positioning of cartilage in certain areas of the PCA muscle. Considering its size, it being considerably bigger than the c. laterale, such control must be divided into a set of different steering units to ensure stability. Due to the fact, however, that supramysial surface electrodes detect more EMG activity from the surface of the muscle than intramuscular array electrodes, it must be concluded that the EMG records will differ. In case of the surface electrodes, there are two rows of electrode surfaces located next to each other on the muscle, whereas the array electrode surfaces are positioned one behind the other inside the muscle. This might explain the significant differences between the electrode types in the EMG pattern comparison, since the individual channels of the surface electrode might diverge more strongly from the pattern. These conclusions might also be in accord with the c laterale EMG results. Since the c. laterale is much narrower and thinner than the c. longum, both electrode types are forced to cover approximately the same muscle area. In addition, the general characteristics of the c. laterale as antigravity muscle must be considered. Due to the fact that the animals are of similar weight, this function and its coordination are less laborious or individual. That is why also the control may be organized in a simpler way. The registered amplitude patterns of both electrode types are thus very similar for the c. laterale. There were notwithstanding significant differences in the amplitudes, which probably resulted from the deeper position of the intramuscular array electrodes inside the muscle belly. In consideration of the relatively small diameter of the c. laterale it can be assumed that even small depth variations in the electrode position can have an effect on the measured EMG amplitudes. Due to the similar proportions of the PCA muscle, this is also to be expected when measuring in the human PCA muscle.

The bipolar electrodes previously used for laryngeal stimulation do not meet the physiological and clinical requirements either in terms of their dimensions or the arrangement of the electrodes and their material properties [3,18,19]. The work published so far on clinical use reports on electrode corrosion and unwanted stimulation of neighbouring muscles. The electrodes used by Zealear et al. in the posterior cricoarytaenoideus muscle (PCA muscle) of dogs also showed channel failures [20]. The supramysial surface grid-electrodes used in this study causes silver corrosion of the electrode surfaces. Measurements over the electrode surface are only possible for a few days. Silver can also cause allergies and inflammation in patients. However, the main problem is the difficult anatomical position of the PCA muscle. This electrode type cannot be used on the human larynx. The intramuscular array electrodes resolves the following limitations: biocompatibility, electrode life span, suitable configuration for larynx stimulation, and EMG measurement.

## 5. Conclusions

The results of both electrode types generally show the alterations in the amplitude level and amplitude pattern of the triceps brachii muscle during movement. When evaluating the details, small differences can be detected between the EMG signals of both electrode types which, however, can be explained physiologically. The fact that there were differences between the signals indicates that the intramuscular electrode may be applied for a detailed analysis of the human larynx. By means of the EMG signals it should be possible to evaluate the PCA muscle function and to detect changes over time.

The results of the present study are promising. New insights on the reinnervation of the PCA muscle can be expected in practice. It is also imaginable that the resulting new EMG measurement method promotes the definition of a more detailed diagnostics which could result in innovating methods of treatment. Finally, the data obtained may also help to optimize stimulation parameters for the larynx pacemaker.
